# Case Report: Primary cardiac atypical lipoblastoma in an adolescent female

**DOI:** 10.3389/fmed.2026.1854633

**Published:** 2026-07-03

**Authors:** Liangjing Lyu, Yuemei Hu, Shun Dai, Yongliang Li, Weiwu Yao

**Affiliations:** 1 Department of Radiology, Tongren Hospital, Shanghai Jiao Tong University School of Medicine, Shanghai, China; 2 Department of Pathology, Tongren Hospital, Shanghai Jiao Tong University School of Medicine, Shanghai, China

**Keywords:** cardiac tumors, case report, CT, lipoblastoma, pericardial tumor

## Abstract

Primary cardiac lipoblastoma is a rare benign tumor of embryonic adipose tissue, typically occurring in infants and young children. This case report describes a 15-year-old female with an atypical lipoblastoma in the pericardial atrioventricular groove—an unprecedented age and location, with imaging features not previously reported. CT showed a hypodense mass without macroscopic fat density, indicating the absence of typical mature adipose tissue, and a slender right coronary artery traversed the lesion without invasion. Postoperative pathology confirmed atypical lipoblastoma. This case illustrates that lipoblastoma may lack macroscopic fat on CT due to immature adipocytic differentiation, and that vascular traversal—as seen with the coronary artery in this case—is a useful imaging clue that may aid radiologists in narrowing the differential diagnosis.

## Introduction

Primary cardiac tumors are exceedingly rare, with an estimated incidence of 0.001–0.3% (1, 2), and more than 90% of them are benign. Nevertheless, even histopathologically benign tumors may lead to significant clinical manifestations due to local invasion or compression of adjacent cardiac structures, potentially compromising cardiac function or disturbing the conduction system. In children, the most common primary benign cardiac tumors (3, 4) are, in descending order, rhabdomyoma, fibroma, and hemangioma.

Lipoblastoma is a benign neoplasm of embryonic adipose tissue that most commonly involves the soft tissues of the extremities. Cardiac localization is extremely rare, with only isolated case reports describing involvement of the pulmonary valve, right atrium, and left ventricle (5–9). The diagnosis relies on histopathology and immunohistochemistry, with molecular testing playing a key role in excluding malignant mimics such as liposarcoma. However, to date, few reports have provided comprehensive multimodality imaging findings or detailed radiological-pathological correlation for cardiac lipoblastoma.

Here, we report a case of lipoblastoma in an adolescent female arising from the pericardial atrioventricular groove. To the best of our knowledge, this specific location in an adolescent patient has not been previously described. Moreover, the imaging features of our case differ distinctly from those reported in previous cases. Therefore, this case report provides distinctive imaging characterization of this rare entity and highlights diagnostic clues that may aid radiologists in their differential diagnosis.

## Case presentation

A timeline of the clinical course is provided in Figure 1. A 15-year-old adolescent female presented with 5 years of episodic, self-resolving chest tightness and pain, unrelated to physical activity. She had no prior cardiac history. Physical examination revealed no precordial bulge, thrills, or pericardial friction rub. Heart sounds were normal with a regular rhythm and no pathological murmurs. Mild jugular venous fullness was noted, and percussion suggested mild cardiomegaly. A routine chest X-ray 2 months before admission revealed a mediastinal mass. Electrocardiography showed sinus bradycardia with arrhythmia (heart rate 50 bpm on ECG) and right-axis deviation (QRS axis 105°, normal range −30° to 90°).

**Figure 1:**
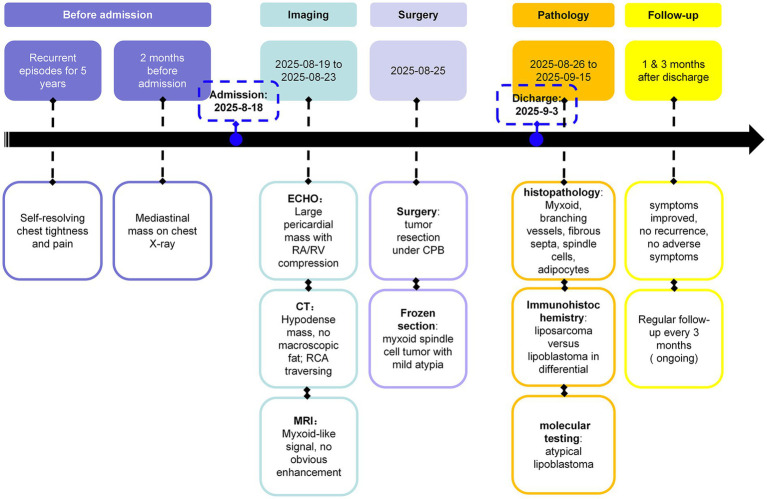
Clinical timeline of the patient’s presentation, imaging, surgery, pathology, and follow-up. Echo, echocardiography; RA, right atrium; RV, right ventricle; RCA, right coronary artery.

Preoperative echocardiography showed a large pericardial mass compressing the right atrium and right ventricle, with tricuspid valve dislocation, inferior vena cava dilation, small-to-moderate pericardial effusion, and mild mitral regurgitation.

Contrast-enhanced CT demonstrated a large, hypodense pericardial mass with homogeneous density and no macroscopic fat density (indicating absence of typical mature adipose tissue). The mass caused marked compression of the right atrium and localized compression of the right ventricle and superior vena cava. It was mostly well defined but indistinct from the pericardium. A slender right coronary artery traversed the mass without invasion (Figure 2). Cardiac MRI showed myxoid-like signal without obvious enhancement, though the mass was not fully captured due to a left-ventricular functional protocol.

**Figure 2:**
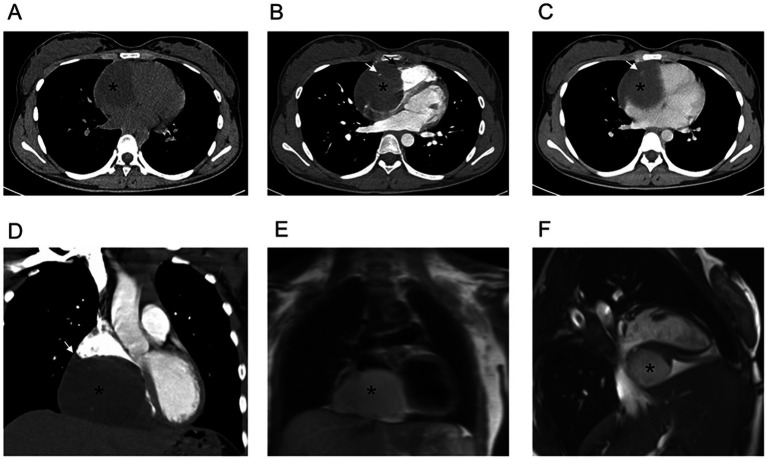
CT and cardiac functional MRI of lipoblastoma. Non-contrast CT **(A)** shows a hypodense pericardial mass (asterisk, 15 HU) compressing the right atrium. Arterial **(B)** and venous **(C)** phases show mild enhancement (24–25 HU); the right coronary artery (white arrow) traverses the mass naturally, and the mass adheres to the pericardium (black arrow), compressing the right atrium and interatrial septum. Coronal reconstruction **(D)** demonstrates the mass protruding into the right heart with mass effect and an acute angle to the pericardium. Cardiac MRI: black-blood coronal T2-weighted image **(E)** and cine left ventricular long-axis view **(F)** both show the mass as hyperintense.

Given the patient’s refractory symptoms and imaging findings, surgical intervention was indicated.

Intraoperatively, a large, yellowish, soft, completely encapsulated tumor arising from the right atrioventricular groove was found. Due to high bleeding risk, cardiopulmonary bypass (CPB) was performed. The tumor capsule was incised, revealing a gelatinous mass with septations and feeding vessels. Partial resection was performed; frozen section suggested a myxoid spindle cell tumor with mild atypia. The tumor was gradually dissected while the right atrial wall defect was repaired. Complete removal was deemed difficult at the right atrioventricular groove; a small residual tumor was left, and meticulous hemostasis was achieved. The procedure was uneventful (CPB time 70 min). Reinfusion included 400 mL of machine blood and 200 mL of autologous blood.

## Pathology findings

Histopathology revealed a myxoid-rich spindle cell neoplasm with scattered lipoblasts, a myxoid background containing thin-walled branching vessels, fibrous septa dividing the tumor into lobules, and adipocytes at various stages of differentiation. Some areas showed hypercellularity and atypical cells with bizarre hyperchromatic nuclei. Immunohistochemistry demonstrated S100 positivity, diffuse strong p16 positivity, and P53 mutant-type expression, with a Ki-67 proliferation index of 10–15%; SMA was focally positive, while desmin, CD34, MyoD1, AE1/AE3, MUC4, and ALK were negative. Molecular testing was negative for MDM2 and CDK4 amplification. These findings confirmed the diagnosis of atypical lipoblastoma. Representative histopathological and immunohistochemical images are shown in Figure 3.

**Figure 3:**
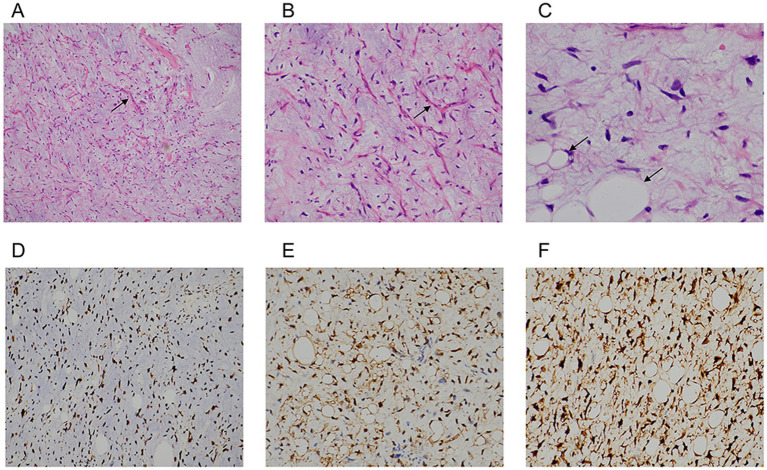
**(A)** Microscopy at ×40 shows a myxoid background with thin-walled branching vessels (arrows) and fibrous septa dividing the tumor into lobules. **(B)** At ×100, delicate branching vessels (arrows) are seen within a myxoid stroma, with sparsely arranged spindle cells showing hyperchromatic, stellate nuclei. **(C)** At ×200, hyperchromatic spindle cells with a fusiform shape are observed; mitotic figures are rare, and adipocytes at various stages of differentiation (univacuolar and multivacuolar forms, arrows) are present. **(D)** Immunohistochemistry at ×100 reveals strong nuclear P53 positivity, suggesting CDKN2A loss. **(E)** Immunohistochemistry at ×100 shows strong cytoplasmic S-100 positivity, supporting adipocytic differentiation. **(F)** Immunohistochemistry at ×100 demonstrates diffuse strong p16 positivity, raising concern for higher aggressive potential or dedifferentiation risk. However, subsequent molecular testing was negative for both MDM2 and CDK4 amplification.

## Follow-up

The patient’s symptoms markedly improved, and she was discharged 9 days later. At one and 3 months of follow-up, she had no adverse symptoms, and echocardiography showed no recurrence. Regular follow-up every 3 months was advised.

## Discussion

Lipoblastoma is a benign neoplasm arising from embryonic white adipose tissue, predominantly occurring in children under 3 years of age. Our patient is a 15-year-old adolescent female with a five-year symptom history that seems to have begun before age ten and followed an indolent course. The age of our patient warrants specific comment. In a series of 59 lipoblastoma cases, age at diagnosis ranged from 3 months to 16 years, with 90% occurring before 3 years of age and only 10% at 10 years or older (10). Our case of a 15-year-old adolescent therefore represents an exceptionally rare age presentation for cardiac lipoblastoma. The tumor most commonly involves the soft tissues of the extremities (11, 12); cardiac localization is extremely rare, with only isolated reports involving the pulmonary valve or atrium.

Standard treatment for lipoblastoma is complete surgical resection. Although it is a benign tumor, local recurrence rates of 19–25% (12) have been reported. Therefore, regular postoperative follow-up is warranted to monitor for potential recurrence.

Intraoperatively, the tumor arose from the pericardial atrioventricular groove and appeared grayish-yellow and myxoid. CT demonstrated hypodense (fluid-like) attenuation rather than typical macroscopic fat density, with mild enhancement—likely due to a prominent myxoid component and immature adipocytic differentiation, which differs from previously reported cases. Notably, CT clearly showed the right coronary artery traversing the mass without invasion, a key finding suggesting soft consistency and indolent behavior. A limitation is that cardiac MRI was performed for ventricular function rather than dedicated tumor imaging, limiting characterization of the mass. A dedicated tumor protocol—including DIXON for detecting subtle or intracellular fat, which may indicate adipocytic differentiation even in the absence of macroscopic fat, diffusion-weighted imaging for tissue characterization, and contrast-enhanced sequences for assessing vascularity—would have provided additional valuable information regarding tumor composition. The mild jugular venous fullness noted on physical examination was consistent with the echocardiographic finding of inferior vena cava dilation, reflecting elevated right atrial pressure due to mass compression. The absence of significant murmurs can be attributed to the soft consistency of the tumor and the absence of significant valvular dysfunction.

The imaging features of the present case differ significantly from those of common pediatric cardiac tumors (e.g., rhabdomyoma, fibroma, and hemangioma). Given the right coronary artery traversing the mass without invasion and the homogeneous hypodense attenuation on CT, the differential diagnosis for a tumor in the right atrioventricular groove primarily includes adipocytic tumors (12), lymphangioma, and lymphoma.

Liposarcoma (13, 14), the most common malignant adipocytic tumor, typically occurs in adults and shows heterogeneous density with solid enhancement and aggressive features. However, myxoid-rich liposarcoma may occasionally be difficult to differentiate from our case; in-phase/opposed-phase MRI can help detect small or immature fatty components, though definitive diagnosis relies on molecular pathology (negative MDM2/CDK4 amplification in our case confirmed atypical lipoblastoma). Cardiac lymphangioma (15) is also extremely rare, predominantly affects children, and is usually asymptomatic and indolent, typically showing no enhancement with a well-defined capsule—distinguishing it from our case. Lymphoma (16) mainly occurs in middle-aged and older adults; although often homogeneous and hypovascular, its attenuation is higher (soft tissue-like) than the fluid-like density seen in our case, and it typically enhances more prominently.

## Conclusion

This case of cardiac lipoblastoma in a 15-year-old adolescent is exceptional in both age and imaging phenotype. Both radiologists and surgeons should be aware that adipocytic tumors may manifest as non-fatty, fluid-like attenuation on CT when fatty components are immature or myxoid-rich, and that vascular traversal is a valuable sign for narrowing the differential diagnosis. Although MRI provided limited information here as it was performed for functional assessment rather than dedicated tumor imaging, such a protocol would be recommended for future cases to provide more comprehensive characterization. The diagnosis was ultimately confirmed by histopathology, immunohistochemistry, and molecular testing, with negative MDM2/CDK4 amplification. This case highlights that cardiac lipoblastoma should be considered in adolescents, and that vascular traversal is a valuable imaging sign to recognize.

## Data Availability

The original contributions presented in the study are included in the article/supplementary material, further inquiries can be directed to the corresponding authors.
